# Fusion of a proline-rich oligopeptide to the C-terminus of a ruminal xylanase improves catalytic efficiency

**DOI:** 10.1080/21655979.2022.2061290

**Published:** 2022-04-20

**Authors:** Ruyue Dong, Xiaoqing Liu, Yaru Wang, Xing Qin, Xiaolu Wang, Honglian Zhang, Yuan Wang, Huiying Luo, Bin Yao, Yingguo Bai, Tao Tu

**Affiliations:** a State Key Laboratory of Animal Nutrition, Institute of Animal Sciences, Chinese Academy of Agricultural Sciences, Beijing, China; b Biotechnology Research Institute, Chinese Academy of Agricultural Sciences, Beijing, China

**Keywords:** Xylanase, catalytic efficiency, enzyme engineering, C-terminus fusion

## Abstract

Xylanases are widely used in the degradation of lignocellulose and are important industrial enzymes. Therefore, increasing the catalytic activity of xylanases can improve their efficiency and performance. In this study, we introduced the C-terminal proline-rich oligopeptide of the rumen-derived XynA into XylR, a GH10 family xylanase. The optimum temperature and pH of the fused enzyme (XylR-Fu) were consistent with those of XylR; however, its catalytic efficiency was 2.48-fold higher than that of XylR. Although the proline-rich oligopeptide did not change the enzyme hydrolysis mode, the amount of oligosaccharides released from beechwood xylan by XylR-Fu was 17% higher than that released by XylR. This increase may be due to the abundance of proline in the oligopeptide, which plays an important role in substrate binding. Furthermore, circular dichroism analysis indicated that the proline-rich oligopeptide might increase the rigidity of the overall structure, thereby enhancing the affinity to the substrate and catalytic activity of the enzyme. Our study shows that the proline-rich oligopeptide enhances the catalytic efficiency of GH10 xylanases and provides a better understanding of the C-terminal oligopeptide-function relationships. This knowledge can guide the rational design of GH10 xylanases to improve their catalytic activity and provides clues for further applications of xylanases in industry.

## Graphical Abstract

**Figure:**
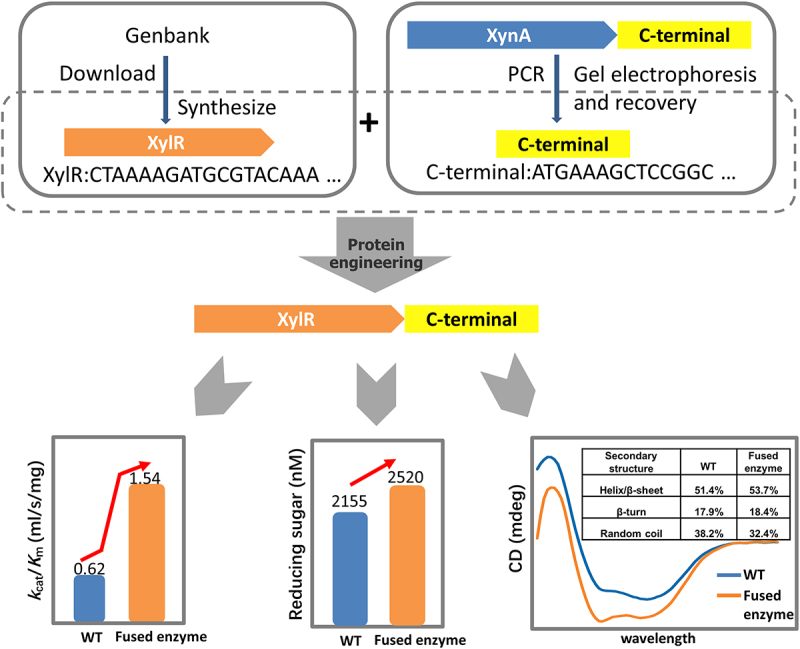

## Highlights

- A proline-rich oligopeptide was fused to the C- terminus of XylR.
- The optimum temperature and pH, and hydrolysis mode of the fused enzyme did not change.
- The oligopeptide increased the catalytic efficiency by 2.48 fold.
- The oligopeptide affected the secondary structure of fused enzyme.

## 1. Introduction

Xylan is the primary component of plant hemicellulose, as it accounts for 7–30% of the dry weight of plant cell walls [1]. The hydrolysis of xylan into xylose and oligosaccharides is mainly catalyzed by endo-β-1,4-xylanases (EC 3.2.1.8). Sequence homology and hydrophobic cluster analyses of the catalytic domain have revealed that most xylanases are members of the glycoside hydrolase (GH) families 10 and 11 [2,3]. In addition, a few enzymes with xylanase activity are found in the GH5, 7, 8, 16, 26, 30, 43, 52, and 62 families [4]. The structure, mechanism of action, and substrate specificity of xylanases differ depending on the source of each enzyme [5].

Xylanases have shown great potential for application in the animal-feed, food, paper, and fuel industries [6]. For example, xylanases can convert pretreated biomass into fermented sugars that can be used for bioethanol production [7,8]. As the world’s leading renewable energy source, bioethanol can mitigate the over-reliance on fossil fuels and contribute to global energy security [9–11]. The efficient enzymatic hydrolysis of lignocellulosic feedstocks is frequently a bottleneck in industrial applications. Recent research has shown that cheap agricultural wastes, such as wheat bran and corncob, have great potential as carbon sources for the production of biomass-degrading enzymes [12,13]. Furthermore, some cost-effective, sustainable, and high-efficiency recovery tools, such as liquid biphasic flotation, are widely used in the large-scale production of high-purity xylanases [14].

The catalytic activity and stability of the xylanases derived from microorganisms are far from meeting the needs of industrial applications [15]. For example, the xylanases that are added to animal feed should have high catalytic activity at the animal body temperature to adapt to the digestive tract conditions, as well as good thermal stability to withstand the high temperature of feed processing [16,17]. Recent studies have identified the C-terminus of xylanases as a functional hot spot that can be modified owing to the high flexibility of its structure [18]. For instance, introducing interactions at the C-terminus or deleting the terminal structure increases the thermostability of the enzyme [19–21].

In previous studies, we identified a high-transcription xylanase gene *xynA* from the sheep rumen [22]. Peptide sequence analysis determined that XynA has a proline-rich oligopeptide at its C-terminus, in addition to a complete GH10 catalytic domain. In the present study, we aimed to reveal the function of this extra C-terminal oligopeptide by fusing it to another GH10 family xylanase, XylR, derived from *Nelore* rumen. XylR is suitable for use in industrial environments because of its tolerance to NaCl and organic solvents; however, its catalytic performance is not outstanding [23]. Through a series of biochemical experiments, we found that introducing the proline-rich oligopeptide improved the catalytic efficiency of XylR-Fu and the release of oligosaccharides from beechwood xylan. In addition, circular dichroism analysis was used to evaluate the function of this C-terminal oligopeptide, which can contribute to the understanding of the possible mechanisms underlying the improvement of catalytic efficiency. Our findings provide a new direction for improving the catalytic efficiency and industrial performance of xylanases.

## 2. Materials and methods

### 2.1 Genes, plasmids, strains and chemicals

The *xynA* gene is derived from the sheep rumen (GenBank accession number JX154664) [22]. The *xylR* gene was synthesized according to the codon preference of the *Escherichia coli* expression systems (GenBank accession number: ACN78954.1; Fig. S1). The expression vector pET-28a(+) (Invitrogen, Carlsbad, CA) was used for exogenous gene expression, whereas *E. coli* XL10 and BL2 (DE3) (Vazyme, Beijing, China) were used for plasmid amplification and protein expression, respectively. Beechwood xylan, xylose, and the xylooligosaccharide standards (xylobiose, xylotriose, xylotetraose, xylopentaose, and xylohexaose) were purchased from Sigma (St. Louis, MO). The other chemicals used in this research are of analytical grade and commercially available.

### 2.2 Sequence analysis

SnapGene software (https://snapgene.com) was used for nucleotide sequence analysis and assembly. The alignment of protein sequences was performed using the BLASTp tool in the NCBI database (http://www.ncbi.nlm.nih.gov/BLAST/).

### 2.3 Construction, expression, and purification of the fused enzyme

The C-terminal oligopeptide of XynA was amplified according to the sequence alignment analysis and then fused to the C-terminus of the XylR catalytic domain using the primer pair Fu-F/Fu-R (Table S1). The PCR fragment was digested with *Eco*RI and *Not*I and ligated into the pET-28a(+) vector. The resulting plasmids, pET-28a(+)-*xylR* and pET-28a(+)-*xylR-Fu* were then transformed into *E. coli* BL21 (DE3) competent cells. *E. coli* cells were grown at 37°C and 200 rpm until the OD reached 0.6–0.8. The final concentration of 0.1 mM isopropyl β-d-1-thiogalactopyranoside (IPTG) was added to the medium, which was then shaken at 200 rpm (30°C, 22 h) to induce protein expression. Finally, the cells were harvested by centrifugation at 8000 rpm for 10 min.

Subsequently, the cells were suspended in lysis buffer (20 mM Tris-HCl , 500 mM NaCl, pH 7.6, 1 mM phenylmethanesulfonyl fluoride), disrupted by ultrasonic waves, and centrifuged at 12,000 rpm for 1 h (4°C). Proteins were separated by the Ni-NTA affinity chromatography. Buffer A (20 mM Tris-HCl , 500 mM NaCl, pH 7.6, 80 mM imidazole) was used to elute non-target proteins, and buffer B (20 mM Tris-HCl , 500 mM NaCl, pH 7.6, 200 mM imidazole) was used to elute target proteins. The purified protein was collected and analyzed by 12% sodium dodecyl sulfate-polyacrylamide gel electrophoresis. The protein concentration was determined using the Bradford assay (BSA is used as standard).

### 2.4 Enzymatic activity assay, biochemical characterization and kinetic parameters

The xylanase activity was measured using the dinitrosalicylic acid (DNS) method, which detects the release of reducing sugars [22]. One xylanase activity unit (U) was defined as the amount of enzyme that releases 1 μmol of reducing sugars per minute.

The optimal pH value of XylR and XylR-Fu were measured at 37°C in McIlvaine buffer (200 mM sodium phosphate, 100 mM sodium citrate, pH 4.0 to 8.0) for 10 min. To determine the optimal temperature of enzymes, assays were performed in the temperature range of 15–52°C at the optimal pH determined previously. Enzyme-kinetics assays were carried out at 30°C in McIlvaine buffer (pH 6.0) containing 1–10 mg/mL beechwood xylan for 5 min. The *K*_m_ and *V*_max_ values were determined by fitting the data to the Michaelis-Menten equation using the GraphPad Prism (version 8.0) software.

(1) $$V=\frac{V_{max}\left[S\right]}{K_{m}+\left[S\right]}$$

(2) $$k_{cat}=\frac{V_{max}}{E_{t}}$$

where V (μmol/min/mg) means the catalytic rate; *V*_max_ (μmol/min/mg) means the maximum enzyme velocity in the same units as V; *K*_m_ (mg/mL) is the Michaelis-Menten constant; [S] (mg/mL) is the initial concentration of substrate; *k*_cat_ (s^−1^) means the turnover number, the frequency of substrate conversion by each enzyme site at per second; E_t_ (mol) is the molar concentration of enzyme catalytic sites [24].

### 2.5 Hydrolysis mode determination

Enzyme samples (6 μM) were mixed with 80 μg/mL xylobiose, xylotriose, xylotetraose, xylopentaose and xylohexaose in 10 mM McIlvaine buffer (pH 6.0), respectively, and incubated at 30°C for 5 h. Samples (500 μL) were then collected and heated at 100°C for 5 min. After filtering the samples with a 0.22 μm microporous filter, the hydrolyzates were analyzed by high performance anion exchange chromatography on a Dionex CarboPac PA-100 (4 x 250-mm) column (HPAEC-PAD; Sunnyvale, CA) [25].

### 2.6 Analysis of hydrolyzates of xylose from beechwood

Reaction mixtures containing 500 μL of 0.5% (w/v) beechwood xylan and 500 μL enzyme samples (10 μM XylR or XylR-Fu) were incubated for 10 h. After heating at 100°C for 5 min, the reaction mixture was filtered through a 0.22 μm filter. XylR and XylR-Fu hydrolyzates of beechwood xylan were detected using HPAEC-PAD. The substances xylose, xylobiose, xylotriose, xylotetraose, xylopentaose and xylohexaose were used as standards.

### 2.7 Circular dichroism (CD) measurement

The CD spectra of XylR and XylR-Fu were measured on a Chirascan spectropolarimeter (Applied Photophysics, Surrey, United Kingdom) with nitrogen at 25°C. The purified protein was diluted to 0.1 mg/mL with 10 mM phosphate buffer saline (PBS) at pH 7.4. The buffer and the diluted enzymes were measured three times in the far-ultraviolet range of 190 nm–260 nm using a 0.1 cm path-length rectangular quartz cell. The baseline correction was performed by subtracting the buffer spectrum. The results were analyzed by CD Spectra Deconvolution (CDNN) processing software.

## 3. Results

This study aimed to enhance the catalytic efficiency of XylR, a bovine rumen-derived, GH10 family xylanase, by introducing a C-terminal proline-rich oligopeptide and to establish a preliminary mechanism to explain the functioning of this oligopeptide. Our data indicated that the optimum temperatures and pH values, hydrolysis mode, and product type of XylR-Fu were not altered by the introduction of the C-terminal oligopeptide. However, XylR-Fu showed higher catalytic efficiency and released more oligosaccharides from beechwood xylan than that of XylR. CD analysis indicated that the proline-rich oligopeptide increased the rigidity of the enzyme, which may directly enhance the enzyme-substrate affinity and therefore the catalytic efficiency.

### 3.1 Sequence analysis and protein expression

**Figure 1.**
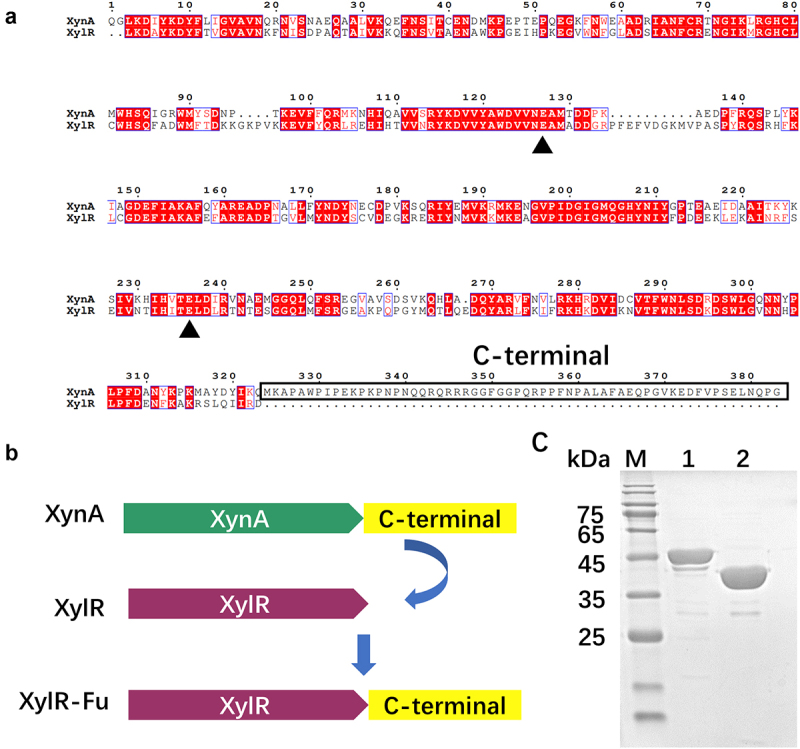
Construction of fused enzyme XylR-Fu based on homologous structure alignment and SDS-PAGE analysis of the purified enzymes. (a) Sequence alignment of XynA and XylR, the conservative catalytic residues are marked with green triangle, and the C-terminal is marked with solid wire box. (b) The construction and schematic structure of the xylanases. The structures and contained domains are shown for XynA, XylR and XylR-Fu. (c) SDS-PAGE analysis of purified XylR (lane 2) and XylR-Fu (lane 1).

The GH10 xylanase XynA contains an extra proline-rich oligopeptide at the C-terminus, in which proline accounts for up to 20% of the 60 total amino acids. XynA had the highest homology with XylR from *Prevotella*, as the UniProt database determined an amino acid sequence identity of 58% (Figure 1(a)). In this study, the extra proline-rich oligopeptide of XynA was fused to XylR (Figure 1(b)). The recombinant plasmids, pET-28a(+)-*xylR* and pET-28a(+)-*xylR-Fu*, were successfully expressed in *E.coli* BL21 (DE3) cells. After induction at 30°C for 20 h, the cell lysate supernatant of XylR-Fu and XylR showed xylanase activities of 12.37 ± 0.10 and 10.90 ± 0.20 U/mL, respectively. The enzyme was purified to > 95% purity using Ni-NTA affinity chromatography. The apparent molecular weights of XylR and XylR-Fu were 39 kDa and 45 kDa, respectively, which were consistent with their theoretical molecular weights (Figure 1(c)).

### 3.2 Enzyme properties and kinetic parameters of XylR and XylR-Fu

**Figure 2.**
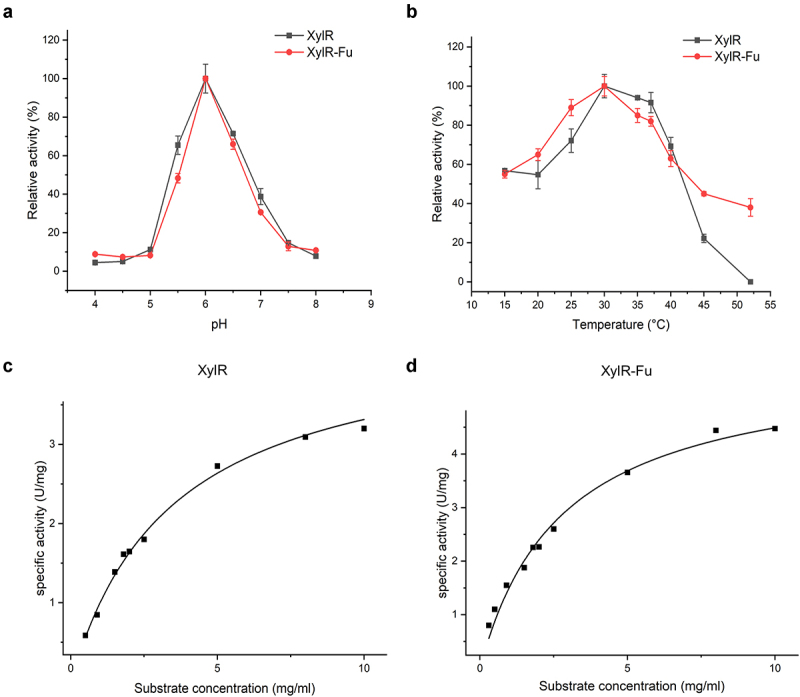
Enzymatic properties of XylR and XylR-Fu. (a) The pH optima of XylR and XylR-Fu; (b) The temperature optima of XylR and XylR-Fu; (c,d) The Michaelis-Menten plots of XylR and XylR-Fu. The kinetic parameters were determined at their optimal conditions.

The properties of purified XylR and XylR-Fu were determined using the beechwood xylan as a substrate. The relative activities of XylR and XylR-Fu did not differ significantly under the same pH. The optimum pH value of both XylR and XylR-Fu was 6.0, and both enzymes maintained more than 40% of their relative activity in the pH range of 5.5–6.5 (Figure 2(a)). Moreover, the two enzymes showed the highest activity at 30°C, as most rumen-derived xylanases, which maintained more than 50% of their maximum activity between 25–40°C (Figure 2(b)). Compared to XylR, the specific activity of the fused enzyme was improved by 1.37 fold (4.93 ± 0.21 vs. 3.60 ± 0.02 U/mg). As shown in Table S2, XylR-Fu had a lower *K*_m_ than XylR (2.79 ± 1.00 vs. 4.79 ± 1.90 mg/mL), indicating that the fused enzyme has a stronger affinity for the substrate than XylR. Notably, the product release rate (*k*_cat_) of XylR-Fu was higher than that of XylR (4.30 ± 0.33 vs. 3.18 ± 4.18 s^−1^), indicating that the fused enzyme was more efficient at substrate turnover than XylR, thereby resulting in a 2.48-fold increase in catalytic efficiency (1.54 vs. 0.62 mL/s/mg) (Figure 2(c, d)).

### 3.3 Analysis of hydrolysis patterns

**Figure 3.**
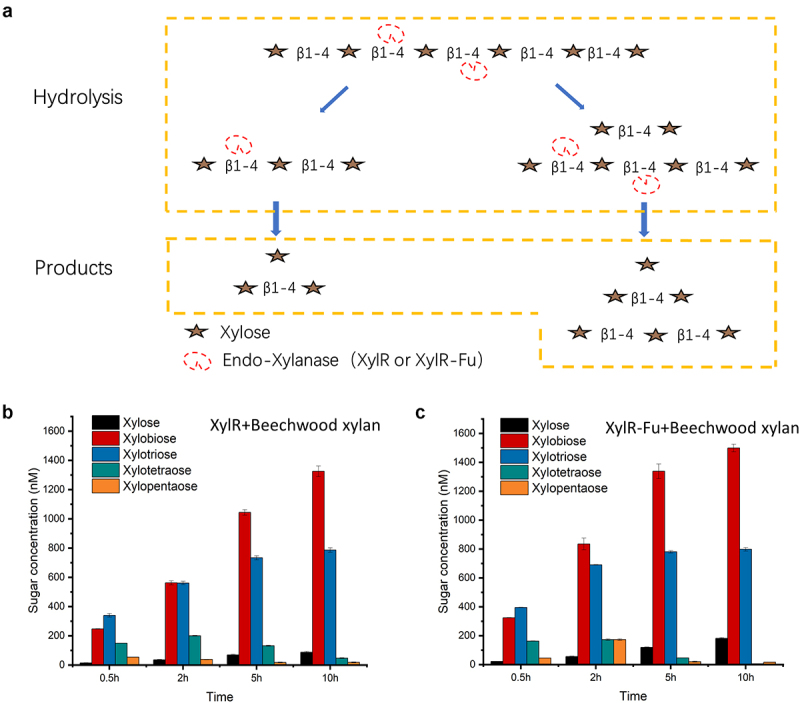
Hydrolysis patterns of two xylanases (XylR, XylR-Fu) and their product profiles after degrading beechwood xylan. (a) Hydrolysis patterns of XylR and XylR-Fu. Taking xylohexaose as an example, xylanase first hydrolyzes xylohexaose into xylobiose, xylotriose and xylotetraose. Xylotetraose continues to be hydrolyzed to xylobiose as well as xylose and xylotriose. In addition, xylotriose is cleaved into xylose and xylobiose. Xylobiose cannot be hydrolyzed. (b) The yield of oligosaccharides by XylR. (c) The yield of oligosaccharides by XylR-Fu.

To explore the hydrolysis patterns of XylR and XylR-Fu, we analyzed the hydrolyzates obtained from the hydrolysis of xylooligosaccharides. The hydrolysis pattern of XylR-Fu was not affected by the fusion of the C-terminal oligopeptide. HPAEC-PAD analysis revealed that both XylR and XylR-Fu completely reduced xylopentaose and xylohexaose to xylose, xylobiose, and xylotriose. Xylotetraose was hydrolyzed to xylobiose and small amounts of xylose and xylotriose. In addition, xylotriose was cleaved into small quantities of xylose and xylobiose. However, xylobiose could not be degraded by either enzyme under these experimental conditions (Fig. S2). Therefore, we concluded that the active sites of XylR and XylR-Fu require at least three xylose residues to hydrolyze the substrate, and both enzymes share the same hydrolysis mode (Figure 3(a)).

### 3.4 Analysis of hydrolysis products

The hydrolysis products of XylR and XylR-Fu on beechwood xylan as substrate were analyzed by HPAEC-PAD from 0.5–10 h. The highest yields of xylotetraose and xylopentaose produced by both enzymes were observed after 2 h. Over time, xylotetraose and xylopentaose were hydrolyzed and were almost completely consumed after 10 h. In contrast, the concentrations of xylose, xylobiose and xylotriose showed an upward trend after 0.5 h and reached their highest concentrations at 10 h (Fig. S3). Notably, the final concentrations of xylose, xylobiose, and xylotriose produced by XylR-Fu were 17% higher than those produced by XylR (2,520 nM vs. 2,155 nM) (Figure 3(b, c)). Although the two enzymes had the same hydrolysis mode, the amounts of xylose, xylobiose, and xylotriose generated by XylR-Fu were greater than those generated by XylR. These results indicate that the degradation efficiency of XylR-Fu was higher and that the fusion of the proline-rich oligopeptide could increase the enzyme catalytic activity.

### 3.5 CD analysis

**Figure 4.**
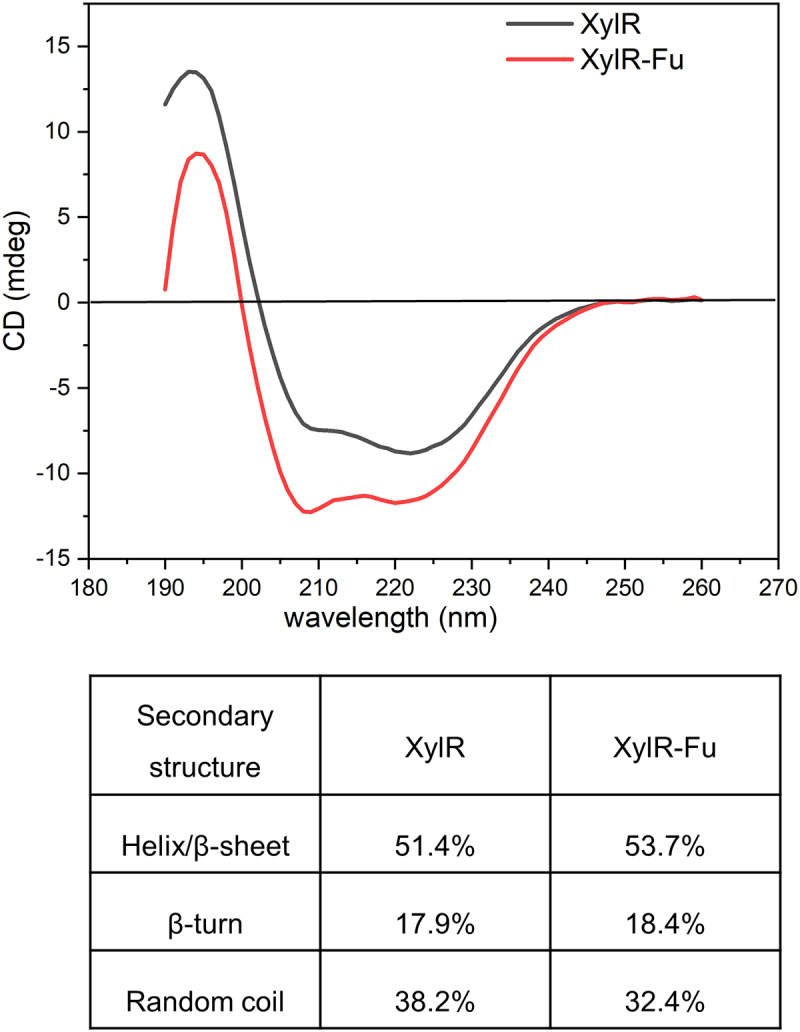
Secondary structural analysis of XylR and XylR-Fu by far-UV CD spectra and their secondary structure contents calculated by CDNN software.

To further analyze the influence of the C-terminal proline-rich oligopeptide on the structure of XylR, CD analysis was performed to detect the secondary-structure changes. The α-helix, β-sheet and random coils had the characteristics of negative CD spectrum bands at ~222 and 208 nm, ~215 nm, and ~195 nm, respectively. As shown in Figure 4, both enzymes showed a maximum value between 192–198 nm, and a minimum value between 210–220 nm, which were consistent with the GH10 xylanase with α-helix and β-sheet characteristics. CDNN software analysis revealed that the α-helix to β-sheet ratio of XylR-Fu was approximately 2.3% higher than that of XylR. Additionally, a gain of 0.5% β-turn and a loss of 5.8% random coils were found in XylR-Fu compared to those in XylR. Therefore, the fusion of the proline-rich oligopeptide altered the secondary structure of the enzyme.

## 4. Discussion

Rumen microorganisms efficiently degrade lignocellulose by secreting lignocellulose-degrading enzymes [26]. Xylanases conduct the first step in the degradation of lignocellulose in animal feed; therefore, xylanases with high catalytic activity are advantageous in animal-feed applications [27,28]. In studies attempting to improve the catalytic activity of xylanases, most of the work were limited to modifying on the catalytic domain [17], with little attention paid to the non-catalytic domain. However, modifications performed by site-directed mutagenesis have not achieved substantial improvements in the catalytic efficiency of xylanases [29].

Currently, the relationship between the non-catalytic domains and catalytic efficiency remains controversial. In previous studies, by addition the non-catalytic domain, the catalytic efficiency of the fusion enzyme was not affected [21,30], or reduced [31–33]. While there are some reports showed that the introduction of the non-catalytic domain can improve the catalytic efficiency of fusion enzymes. For example, the catalytic efficiency of the *Paenibacillus* xylanase, using birchwood xylan as substrate, was increased by 1.5 fold after the introduction of a xylan-binding domain [34]. Furthermore, inserting the submodule C2 from Carbohydrate-binding modules 9_1-2 into the N- and/or C-terminal regions of *Neocallimastix patriciarum* xylanase increased (18–28%) its catalytic activity toward corncob xylanase [35]. In the present study, the proline-rich C-terminal oligopeptide enhanced the catalytic activity of xylanase by 2.48 fold, which is a remarkable effect in this type of studies. Therefore the catalytic efficiency of fusion enzymes is not only related to the amino acid sequences of the enzymes, but also to the properties of the non-catalytic domains.

In this study, the proline-rich oligopeptide at the C-terminus of XynA was fused to the homologous protein XylR to investigate the effects of this oligopeptide on the catalytic activity. This oligopeptide did not change the optimum pH and temperature values or the hydrolysis mode of the enzyme, implying that its existence did not affect the hydrolysis of the substrate by the catalytic domain. The decreased *K*_m_ and increased *k*_cat_ values suggested that XylR-Fu had greater catalytic activity than XylR, which may be due to the interaction between the C-terminal region and N-terminal catalytic domain. This interaction may guide the correct folding of the catalytic domain of xylanases or affect the conformational changes of their active site residues. These changes facilitate the enzyme’s access to the substrate by increasing its affinity for the substrate, reducing the resistance to product release, and increasing the overall enzymatic turnover, thus improving the catalytic efficiency [36].

The amount of xylose, xylobiose, and xylotriose released by XylR-Fu was 17% higher than that those released by XylR when beechwood xylan was used as substrate. These findings further indicate that improving the catalytic efficiency of fused enzymes can more completely hydrolyze beechwood xylan. The high catalytic efficiency and oligosaccharide yield of fused enzymes also show great application potential in the animal-feed industry. For ruminants, approximately 50% of the crude fiber in the feed is digested in the rumen. The improved catalytic efficiency of the fused enzyme can enhance the utilization and absorption of hemicellulose in the rumen. In addition, the increased production of xylooligosaccharides can maintain the balance of the animal gut microbiome, thereby promoting animal health [37].

The composition of the C-terminus of XynA was substantially different from that of the general loop. The proportion of Pro in the oligopeptide (60 amino acids) was as high as 20%, whereas some loops in other enzymes are primarily composed of flexible Gly or have a low Gly/Pro ratio composition [30,38]. The side chain of proline is bound to the backbone amide position to form a distinctive cyclic structure [39,40]. As a -CH2 group replaces the amide proton, proline can only serve as a hydrogen bond acceptor. These characteristics may play a role in peptide bond formation [22,41]. This proline-rich sequence was identified as a non-repetitive proline-rich sequence. PRPs are widely distributed in prokaryotes and eukaryotes, and one of their main roles is binding [42]. We speculate that this C-terminal proline-rich oligopeptide may extend to form a ‘sticky arm’, which may provide a large binding surface and multiple contact points to assist the binding of the catalytic domain to different substrates [43,44].

In fact, proline-rich regions are widely present in prokaryotes and eukaryotes proteins and are one of the common binding motifs in protein-protein interaction domains. For example, proline-rich ligand sequences are more likely to bind to some protein interaction modules, such as Src homology 2 and 3 domains, phosphotyrosine binding domains [45]; while proline-rich proteins in saliva can bind tannins and protect organisms from their anti-nutrients effect [46]. It is worth noting that the role of proline-rich regions in helping enzymes bind to substrates was discovered for the first time.

The proline-rich oligopeptide also contained glutamic acid, which has an anionic carboxylate structure that readily forms hydrogen bonds and salt bridges and has a tendency to form helices. The prediction of the secondary structure of the oligopeptide showed that it formed two helices with a coil structure in-between, which may allow the protein to bind to the receptor without changing its conformation. CD analysis indicated that this proline-rich oligopeptide affected the secondary structure of the xylanase, which was manifested by increased α-helix and β-sheet content and decreased random coil content in the fused enzyme. These conformational changes may increase the rigidity of the entire structure, thereby enhancing substrate affinity and ultimately increasing catalytic activity. However, we could not predict its structure because of the lack of homologs of the proline-rich oligopeptide. Relying solely on the analysis of the primary structure would overlook key information of this region. Therefore, a precise tertiary structure is needed to analyze the structure-function relationship of this C-terminal oligopeptide.

## 5. Conclusion

We have demonstrated a C-terminal oligopeptide could enhance the catalytic efficiency of GH10 xylanases. The analysis of the mechanism underlying this improvement showed that the introduction of this proline-rich oligopeptide not only increased the affinity of the enzyme for the substrate and the rate of product release, but also changed the secondary structure composition by increasing the rigidity of the overall structure. In future studies, we hope to clarify the structural basis of the C-terminus function by analyzing its crystal structure. As the understanding of the structure-function relationship advances, more xylanases could be engineered to achieve better performance in industrial processes.

## Supplementary Material

Supplemental Material
