# Supplementary material for: Fusion of a proline-rich oligopeptide to the C-terminus of a ruminal xylanase improves catalytic efficiency
Source: Bioengineered. 2022 Apr 20;13(4):10482–92. doi: 10.1080/21655979.2022.2061290 (PMC9161913; doi:10.1080/21655979.2022.2061290)
Supplement: Supplemental Material [file KBIE_A_2061290_SM4959.docx]

***Supplementary materials***

**Fusion of a proline-rich oligopeptide to the C-terminus of a ruminal xylanase improves catalytic efficiency**

Ruyue Dong,*^1^* Xiaoqing Liu,*^2^* Yaru Wang,*^1^* Xing Qin,*^1^* Xiaolu Wang,*^1^* Honglian Zhang,*^1^* Yuan Wang,*^1^* Huiying Luo,*^1^* Bin Yao,*^1^* Yingguo Bai,*^1^*^,^* Tao Tu*^1^*^,^*

*^1^*State Key Laboratory of Animal Nutrition, Institute of Animal Sciences, Chinese Academy of Agricultural Sciences, Beijing 100193, China

*^2^*Biotechnology Research Institute, Chinese Academy of Agricultural Sciences, Beijing 100081, China

*Corresponding authors.

E-mail: tutao@caas.cn (T. Tu), baiyingguo@caas.cn (Y. Bai)


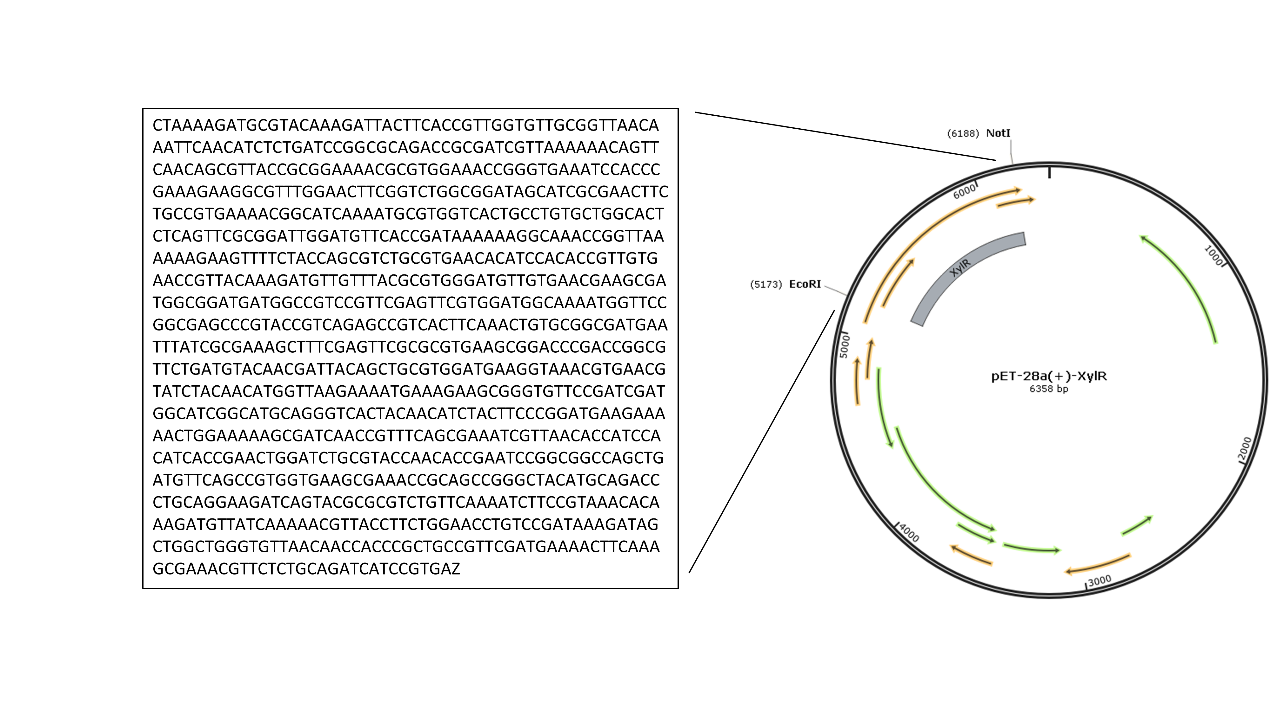


**Fig. S1.** Gene sequence optimized of XylR.

**
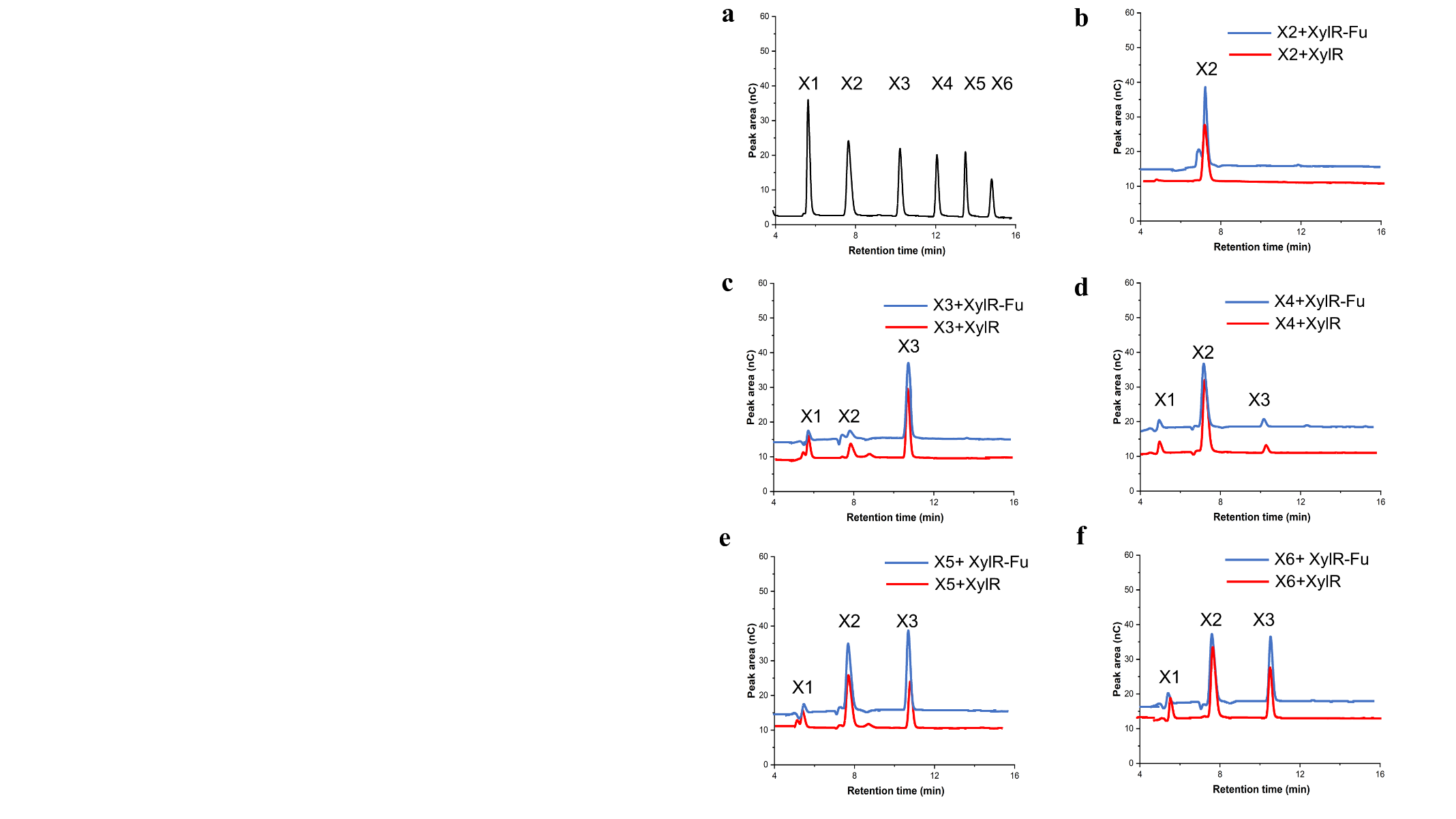
**

**Fig. S2.** Product profile obtained from the hydrolysis of xylooligosaccharides. Enzyme samples (6 μM) were mixed with 80 μg/mL xylobiose, xylotriose, xylotetraose, xylopentaose and xylohexaose, respectively. The reactions were incubated at 30 °C for 5 h. a Standard sugars: xylose (X1), xylobiose (X2), xylotriose (X3), xylotetraose (X4), xylopentaose (X5), and xylohexaose (X6); b X2; c X3; d X4; e X5; f X6.

**
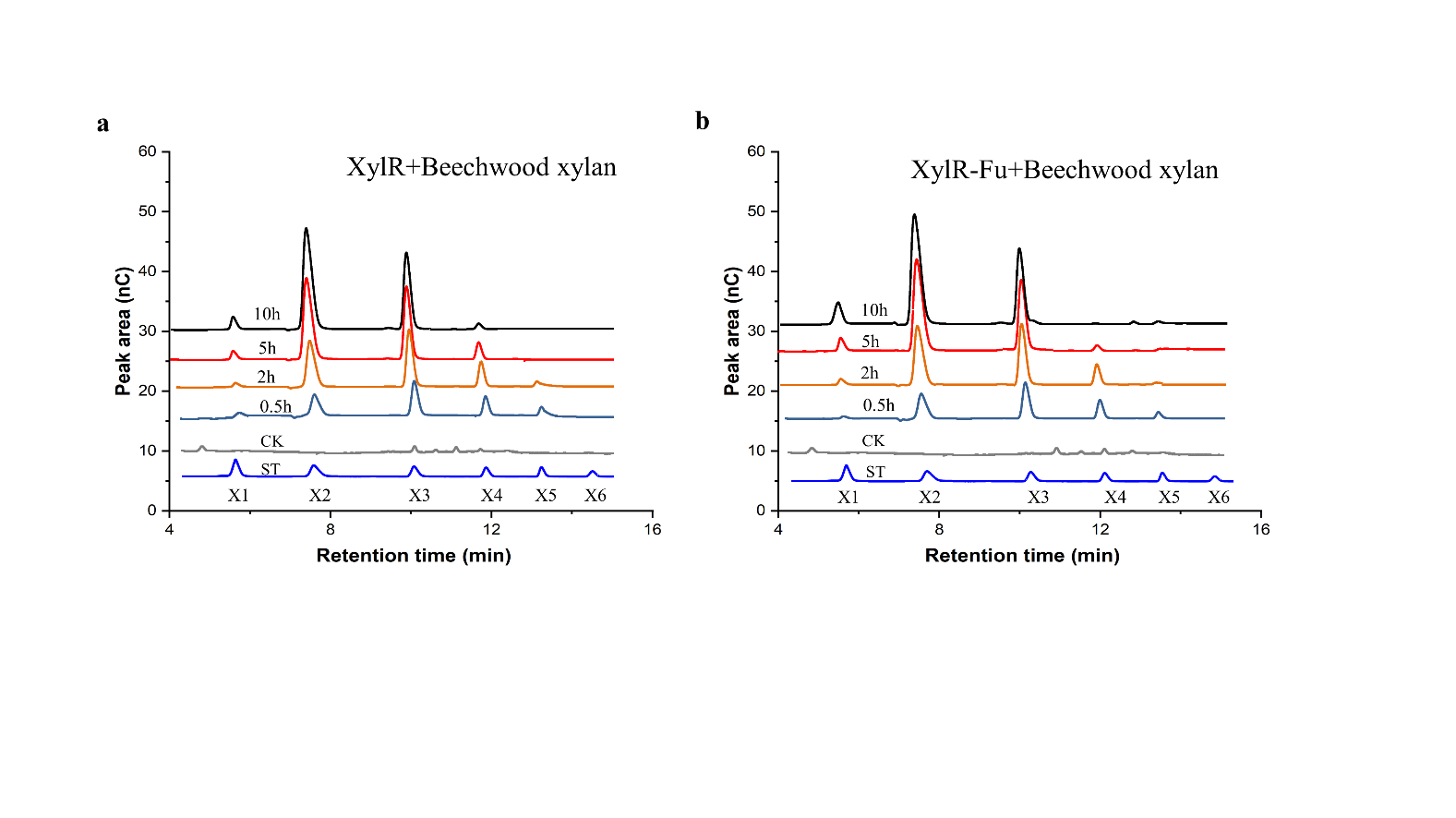
**

**Fig. S3.** Time-course hydrolysis of beechwood xylan by XylR and XylR-Fu. The reactions were conducted under the enzyme's optimal conditions. CK is the blank control without enzymes, and ST is the standard used, which contained xylose (X1), xylobiose (X2), xylotriose (X3), xylotetraose (X4), xylopentaose (X5), and xylohexaose (X6).

**Table S1.** Primers used in this study.

| **Primers** | **Sequences** |
| --- | --- |
| Fu-F | TGCAGATCATCCGTGATATGAAAGCTCCGGC |
| Fu-R | CTCGAGTGCGGCCGCTCCAGGCTGGTTGAG |

**Table S2.** Kinetic parameters of XylR and XylR-Fu.

| **Enzymes** | ***K*_m_**  **(mg/ml)** | ***V*_max_**  **(μmol/min/mg)** | ***k*_cat_**  **(/s)** | ***k*_cat_/*K*_m_**  **(ml/s/mg)** |
| --- | --- | --- | --- | --- |
| XylR | 4.90±1.90 | 4.71±0.70 | 3.18±4.18 | 0.62 |
| XylR-Fu | 2.79±1.00 | 5.74±0.60 | 4.30±0.33 | 1.54 |
